# Osteogenic differentiation of adipose-derived stem cells on dihydroartemisinin electrospun nanofibers

**DOI:** 10.1186/s13036-022-00294-9

**Published:** 2022-06-23

**Authors:** Nazila Shabestani, Hanieh Mousazadeh, Fahimeh Shayegh, Somayeh Gholami, Ali Mota, Nosratollah Zarghami

**Affiliations:** 1 Stem Cell Research Center, Tabriz University of Medical Sciences, Tabriz, Iran; 2 Department of Clinical Biochemistry and Laboratory Medicine, Faculty of Medicine, Tabriz University of Medical Sciences, Tabriz, Iran; 3 Department of Medical Biotechnology, Faculty of Advanced Medical Sciences, Tabriz University of Medical Sciences, Tabriz, Iran; 4 Department of Medical Biochemistry, Faculty of Medicine, Istanbul Aydin University, Istanbul, Turkey

**Keywords:** Dihydroartemisinin, Polycaprolactone/Collagen, Nanofibers, Osteoblastic differentiation, Sustained release

## Abstract

**Background:**

Adipose tissue-derived stem cells (ASCs) are promising candidate in stem cell therapies, and maintaining their stemness potential is vital to achieve effective treatment. Natural-based scaffolds have been recently attracted increasing attention in nanomedicine and drug delivery. In this study, Dihydroartemisinin (DHART)-loaded polycaprolactone collagen nanofibers (PCL/Col NFs) were constructed as effective biocompatible scaffolds through adjusting the proportions of hydrophobic/ hydrophilic polymers for enhanced osteoblastic differentiation of human adipose-derived stem cells (hADSCs).

**Results:**

The designed NFs were characterized through FTIR, XRD, TGA, FE-SEM, and tensile testing. DHART-loaded PCL/Col electrospun NFs provide an ideal solution, with the potential of sustained drug release as well as inhibition of drug re-crystallization. Interestingly, inhibiting DHART re-crystallization can improve its bioavailability and provide a more effective therapeutic efficacy. Besides, the data set found through FE-SEM, MTT, PicoGreen, qPCR, and alkaline phosphatase (ALP) assays revealed the improved adhesion and proliferation rate of hADSCs cultured on PCL/Col/DHART (5%) NFs after 14 and 21 days of incubation.

**Conclusions:**

These findings confirmed the potential of the designed NF scaffolds for sustained/controlled release of DHART therapeutic molecules toward bone tissue regeneration and engineering.

**Graphical Abstract:**

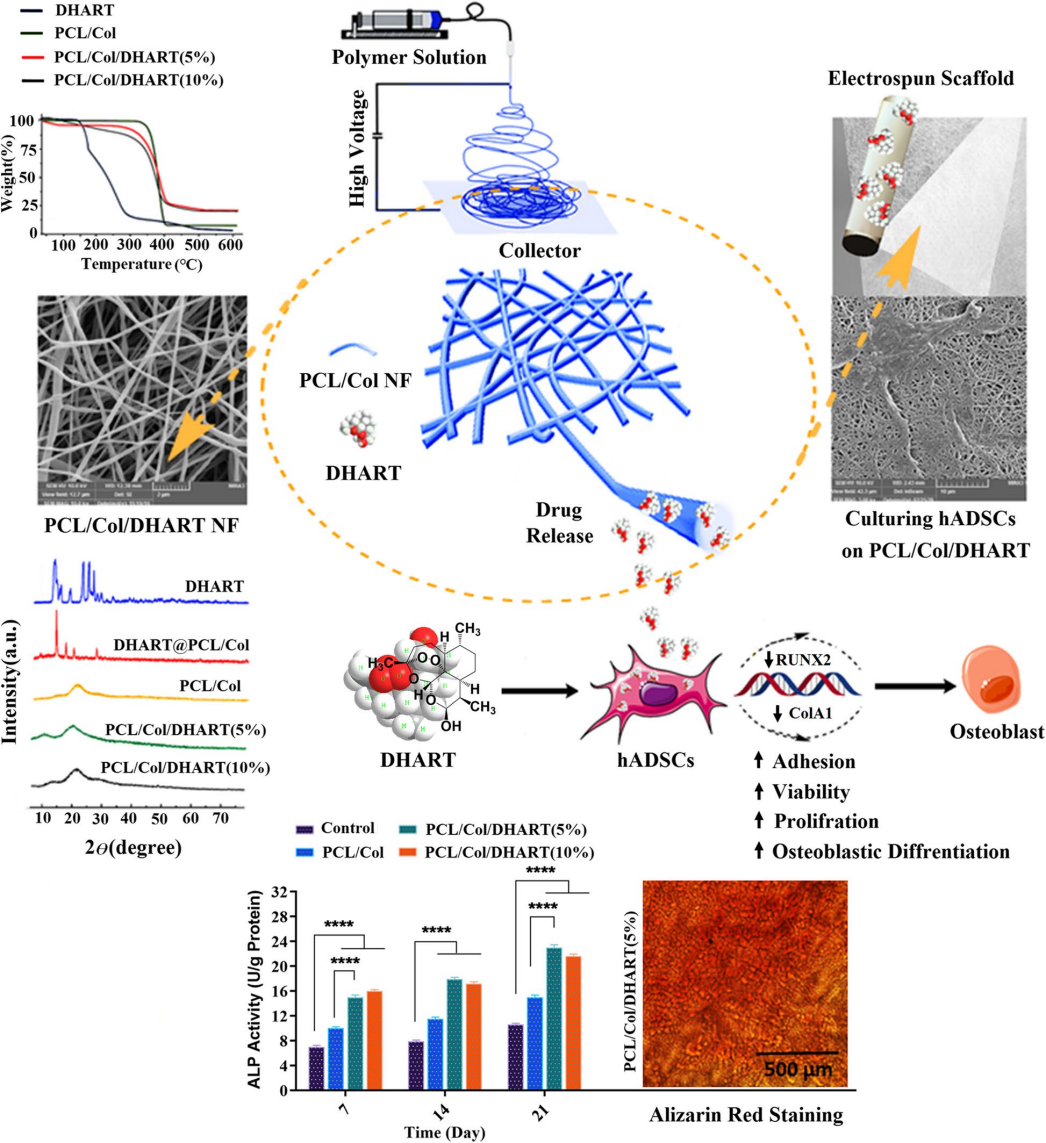

## Introduction

The bone is a highly complex, dynamic, and vascularized tissue that can regenerate itself in a limited area during life. However, large bone disorders induced by trauma, tumors, congenital disorders, and aging are considered the main health problem [1]. Thus, bone grafts or bone substitutes are the most suitable and preferred options for this condition. There have been attempts to regenerate bones through autogenic and allogenic bone grafts, but their applications have been limited by donor-site morbidity, host immune responses, limited availability of grafts, and the risk of disease transmission [2]. Thus, synthetic or natural grafting materials have been developed as bone substitutes for the restoration of bone defects [3].

The combination of scaffolds made from biomaterials, stem cells, and bioactive factors offers a promising platform for bone regeneration [4, 5]. Human adipose-derived mesenchymal stem cells (hADSCs) isolated from lipoaspirate tissue have attracted much attention since they possess multi-lineage capacity, enabling them to differentiate into adipogenic, chondrogenic, myogenic, and osteogenic cells. Therefore, these stem cells have a significant potential for bone regeneration owing to their easy accessibility and availability, high proliferation and differentiation ability, and hypoimmunogenic nature [6, 7].

Tissue engineering also needs biocompatible and porous scaffolds that provide an appropriate microenvironment for the homogenous distribution of the cells and their nutrients without any immune response [8]. A variety of techniques such as electrospinning, phase separation, and self-assembly [9] have been developed to mimic the extracellular matrix as well as its natural structure.

Electrospinning is a promising technique to generate ideal NF scaffolds for bone regeneration. A perfect biomimetic scaffold not only provides favorable physical support but also delivers specific bioactive molecules and stem cells in a three-dimensionally (3D) controlled manner to the damaged tissue [10]. This technique fabricates favorable scaffolds by using a variety of synthetic, e.g., poly (glycolicacid) (PGA), polylactic-*co*-glycolic (PLGA), and poly caprolactone (PCL) and naturally derived biomaterials (e.g., collagen, fibroin) to provide a suitable environment for the cells. These scaffolds can be employed as drug delivery platforms because of their high specific surface area [11–15]. Electrospinning can improve encapsulation efficiency and maintain pharmacological activity by loading pharmaceuticals or biologically active compounds into a polymer [16–18]. Anticancer efficiency of curcumin-loaded mesoporous silica nanoparticles/nanofiber composites for potential postsurgical breast cancer treatment. This process can also decrease the drug burst release by selecting the right drug/polymer/solvent combination [16]. Moreover, the quick-drying procedure of electrospinning facilitates the dispersion of crystalline pharmaceuticals. Hence, electrospun NFs can be used to prevent model drug re-crystallization [19]. Yu et al. reported a series of core–shell NFs that could dissolve and disseminate poorly water-soluble pharmaceuticals while inhibiting drug re-crystallization [20].

Collagen is a good material as an injectable or implanted scaffold for bone regeneration, because of its permeableness, in vivo stableness, and intrinsic biochemical capabilities. The matrix of the bone also contains collagen type 1 (Col1), which is necessary for bone mineralization [3]. Electrospun collagen fibers show high cellular adhesion and growth properties, allowing cells seeded on these scaffolds to differentiate and attach throughout these scaffolds more readily. Due to its weak mechanical qualities and fast breakdown rate, collagen cannot be an appropriate scaffold for the regeneration of bone. These limitations have been overcome by combining collagen with synthetic biocompatible polymers such as polycaprolactone (PCL). PCL is a biocompatible material certified by the FDA with optimum mechanical and degrading properties. Thus, electrospun NF scaffolds for bone regeneration with high cell adhesion and growth characteristics are ideal scaffolds based on PCL/Col composite [21]. PCL's hydrophobic nature could be resolved by using hydrophilic Col. Recently, the researchers synthesized insulin-chitosan nanoparticle-loaded PCL/Col NFs and evaluated their water absorption capacity, surface wettability, microstructure, blood compatibility, mechanical characteristics, and cellular activity. It has been found that PCL/Col NFs have potential medicinal applications [22].

A variety of bioactive compounds such as herbal extracts and flavonoids have been incorporated into NF scaffolds for bone regeneration. Dihydroartemisinin (DHART), one of the active components in Artemisia annua, is categorized into the sesquiterpene lactones family. DHART has shown to be effective against fibrosis, parasite infections, and malaria with little toxicity [23]. In recent years, the effects of DHART have been widely investigated. Besides, DHART exerts anticancer action in human malignancies including hepatomas through activating caspase-independent and caspase-dependent cell death pathways. Komaki and Zhou reported that DHART inhibited osteoporosis and osteoclast formation induced by estrogen deficiency [23]. Besides, Cao reported that DHART had a minor impact on the hypertrophic and chondrogenic differentiation of MSCs. Recently, the function of DHARTs on hMSC proliferation and osteogenic differentiation, as well as the inherent molecular mechanisms, was studied. Based on these results and the low toxicity of DHART, it may have important applications in clinical settings.

Despite DHART's significant therapeutic efficacy, poor bioavailability due to high water solubility and poor permeability across cell membranes has restricted its clinical application. Electrospun NF scaffolds have shown great promise as a drug delivery platform. Therefore, in the current study, DHART-loaded PCL/Col NFs were manufactured through an electrospinning procedure. The proportions of hydrophobic/hydrophilic PCL/Col polymers were adjusted as DHART carriers, and their morphology and functional properties were examined. The prepared PCL/Col/DHART NF scaffolds inhibited the crystallization of DHART and demonstrated their remarkable promise as a sustained DHART drug delivery system. Furthermore, the efficiency of these electrospun NFs for osteoblastic differentiation was evaluated by detecting stem cell proliferation, calcium secretion, alkaline phosphatase activity, and the expression mRNA levels of kay osteoblast differentiation markers.

## Experimental procedure

### Materials

Polycaprolactone (PCL, MW 80,000), rat tail collagen types I, Dihydroartemisinin (DHART), l-ascorbic acid, dexamethasone, β-glycerol phosphate, and MTT powder were obtained from Sigma Aldrich. Dulbecco's Modified Eagle's Medium (low glucose DMEM), penicillin, streptomycin, trypsin, and fetal bovine serum (FBS) were obtained from Gibco.

### Instrumentation

NF scaffolds imaged with FE-SEM (MIRA3 TESCAN, Czech, at 25 kV). The FT-IR spectra were obtained on a Bruker Tensor 270 spectrometer. Thermogravimetric analysis (TGA) was achieved in the temperature range of 100–600 °C using a thermal analysis instrument (SDTQ600). X-ray diffraction (XRD) assay was followed by a Bruker D8 Advance diffractometer using CuKa radiation (λ = 1.542 A˚), as previously reported [24, 25]. The samples were properly cut into 10 mm × 50 mm square strips and were tested by a Testing Machine (1446, Zwick, Germany) with a 10 N load cell and a crosshead speed of 10 mm/min.

### Fabrication of NFs

First, PCL polymer was prepared with ring-opening polymerization of ε-caprolactone monomers [26]. The blend of PCL/Col with a ratio of 80:20 was dissolved into formic acid/acetone with a ratio of 4:1 and stirred at room temperature for 24 h. DHART (5% and 10% concentration) was added into the resulting homogenous solution and stirred for another 24 h at room temperature. Then, the electrospinning solution was vortexed and placed into a standard 5 ml plastic syringe which connected to the stainless steel needle (22 gauge), at the front of the needle was placed collector. During the electrospinning, the parameters were regulated as follows; voltage, the distance between needle and collector, and flow rate were 27–29 kV, 140–170 mm, and 0.25–0.6 ml/h, respectively.

### Degradation assay

For the investigation of degradation properties, the produced NFs inserted on a 24-well plate with 1 mL PBS (pH 7.4) within every well and raised at 37 °C for various time intervals. Then, after each degradation interval, the samples were rinsed and vacuumed dry at room temperature for 24 h. The percentage of reduced weight was evaluated using the equation below. (W0 indicates the fiber weight at the initiation of the degrading experiment, the fraction of fiber weight loss after time t is Wt loss percent, and Wt is the weight of the fiber after time t.

$$\mathrm{Water\;uptake\;capacity}\left(\mathrm{\%}\right)=\frac{w0-wt}{w0}\times 100$$

The samples were cut into a square form (10 $\times$ 50 mm) for tensile testing by a Testing Machine (1446, Zwick, Germany) with a 10 N load cell and a 10 mm/min cross-head speed at ambient temperature as described previously [27].

### Drug release

PCL/Col/DHART (5%) were diluted in PBS and kept at 37 °C for the period of the hydrolysis experiment. At various time points, aliquots were collected, high-speed centrifuged to get supernatant, processed as described previously [28], and UV–Vis measured at 238 nm. The percentage was determined based on the absorbance of the sample at 0 to 21-day vs the primary absorbance.

### MTT Assay

For this assay, circular disks were initially cut from the fibers and sterilized by being exposed to UV for 12 h. These fibers were then rinsed by PBS three times before being seeded into a 24-well plate. hADSCs (passage 3) were purchased from the Pasteur Institute of Iran (Tehran, Iran). The cells were cultured in DMEM enriched with FBS (10%) and penicillin/streptomycin (1%). On the top of the scaffolds, 2 × 10^4^ cells/well were seeded in the 24-well plate. The cells were incubated for four weeks (5% CO_2_, 37 °C). At 1-, 7-, 14-, and 21- days following being seeded, the cells' metabolic activity was determined via the MTT assay. For this purpose, the cells embedded on the NFs were washed with PBS twice before being incubated with an MTT reagent (100 μL) for 4 h at 37 °C. After discarding the medium, formazan crystals were dissolved by adding DMSO. Dissolved formazan (100 μL) was then translocated into a 96-well plate, and the absorbance of the supernatant was then measured at 570 nm using an ELIZA reader (Multiskan MK3, Thermo Electron Corporation, USA). The mean SD (*n* = 3) results were displayed for each experiment.

### PicoGreen assay

The cell proliferation percentage of hADSCs planted on the NFs was measured by a PicoGreen examination (Invitrogen Ltd., Paisley, UK) after 1-, 7-, 14-, and 21- days incubation. For this purpose, per well scaffold/cell received 500 mL of cell lysis solution (Triton X-100 (0.1%), sodium dodecyl sulfate (1%), Tris (10 mM), and EDTA (50 mM)), which was then frozen and thawed three times and stored at -70 °C. Following the approach, the DNA level of each well was determined by mixing each sample (10 µL) with a PicoGreen reagent (200 µL). Finally, the samples were placed in an incubator for 10 min in the dark, and the change in absorbance was evaluated using a microplate reader (Synergy HT, λem: 520–540 nm, λex: 480–500 nm, BioTek).

### Cell adhesion assay

For the study of the cell adhesion manner of the treated NF scaffolds, the cells were plated onto the scaffolds. After incubation for 14-, 21- days, these samples were rinsed with PBS solution three times and then fixed by glutaraldehyde (2.5%) at room temperature for 3 h. The scaffolds were washed with PBS solution and then dehydrated using a range of ethanol concentrations (50%, 70%, 80%, 95%, and 100%) for 10 min each. Finally, the samples were rinsed with PBS solution and were dried with ethanol for FE-SEM analysis.

### Osteoblastic differentiation assay

Cell lines were seeded in an osteogenic induction medium consisting of high glucose DMEM, dexamethasone (10^–7^ M), β-glycerophosphate (10 mM), and ascorbic acid 2-phosphate (0.5 mM) in the 24-well plates to determine the activity of alkaline phosphatase (ALP). After 7, 14, and 21 days of culture, an ALP microplate test kit (Nanjing Jiancheng Bioengineering Institute, Nanjing, China) was used to evaluate ALP activity. The samples were fixed with formalin (4%). Then, they were stained with alizarin red (0.1%) and examined using a stereomicroscope after 21 days of incubation to assess the quantity of calcium accumulated on cell/scaffold constructs (Olympus SZXs-TR30, Japan).

### Real-time analysis

Adipose-derived stem cells (2 $\times$ 10^4^) were seeded in 6-well plates and cultured with 2 mL of osteogenic medium. After incubation for 7-, 14-, and 21- days, cells were lysed by Trizol reagent. According to the manufacturer's process, total RNA was extracted using an RNeasy mini kit (Qiagen, USA) and measured by using a NanoDrop (ThermoScientific, USA). For the cDNA synthesis, an appropriate proportion of RNA was taken from each sample and reverse transcribed through RevertAid First-strand cDNA synthesis Kit (Fermentas, St Leon-Rot, Germany). A Real-Time PCR system was followed using Syber Green Master Mix (BioMolecular Systems, Australia) for the target genes relating to the stated primers as described previously [29].

### Statistical analysis

GraphPad Prism 8 (GraphPad Software, Inc., La Jolla, CA) was used for statistical analysis. The results were evaluated using a one-way ANOVA Analysis of Variance. All the samples were analyzed in triplicates and the results provide as mean ± standard deviation (SD) for *n* = 3. The *p*-value was used to determine the level of significance. Statistically, *P* value < 0.05 was considered significant.

## Results and discussion

### Scaffold Characterization

DHART-loaded PCL/Col NFs (5%, and 10% (wt/wt%)) were constructed through the electrospinning process under the optimal conditions. Any further addition in DHART amount resulted in the therapeutic compounds precipitate in the solution and the formation of electrospun beaded fibers.

**Fig. 1:**
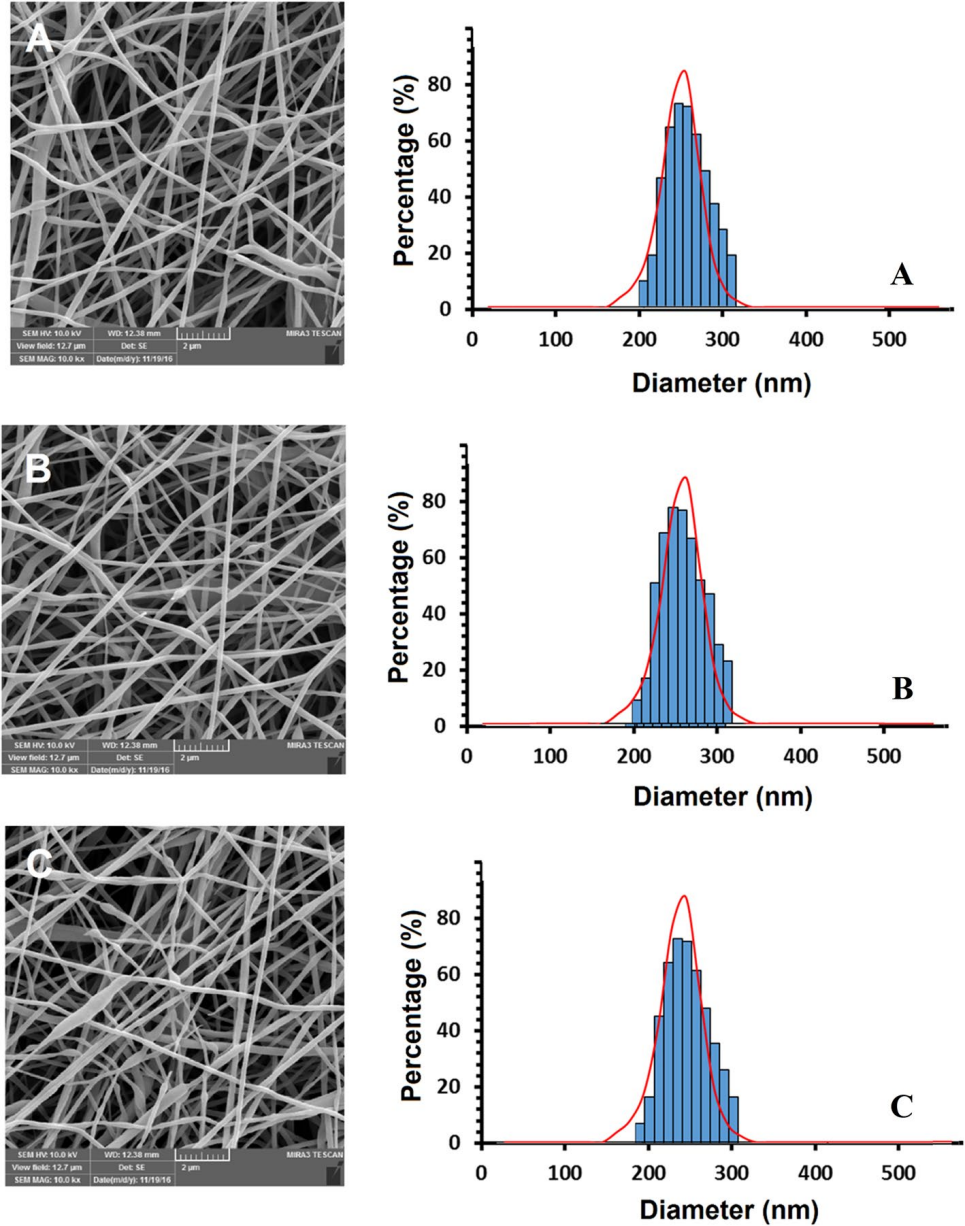
Morphology and diameter distribution of electrospun NFs. FE-SEM images of **A** PCL/Col, **B** PCL/Col/DHART (5%), and **C** PCL/Col/DHART (10%)

According to FE-SEM results, the PCL/Col NFs exhibited smooth and free-bead surfaces with a 250 nm mean size (Fig. 1). Although comparable features to PCL/Col NFs were achieved by encapsulating DHART into PCL/Col NFs, the mean thickness was increased to 320 nm, demonstrating a homogenous distribution of the drug in the polymeric solution and a steady increase in the NF diameter as the drug and polymer are physically blended. These findings are in agreement with those of Hokmabad VR et al. They found that the manufactured PCL-PEG-PCL (PCEC) NFs were bead-free, and smooth fibers with a mean size of 138.851.7 nm while the average thickness of Elaeagnus angustifolia (EA)-loaded PCEC NFs incorporated with 5 wt-%, 10 wt-% and 15 wt-% EA were 180.856.3, 200.4768.5 and 237.2493.6 nm, respectively (1).

**Fig. 2:**
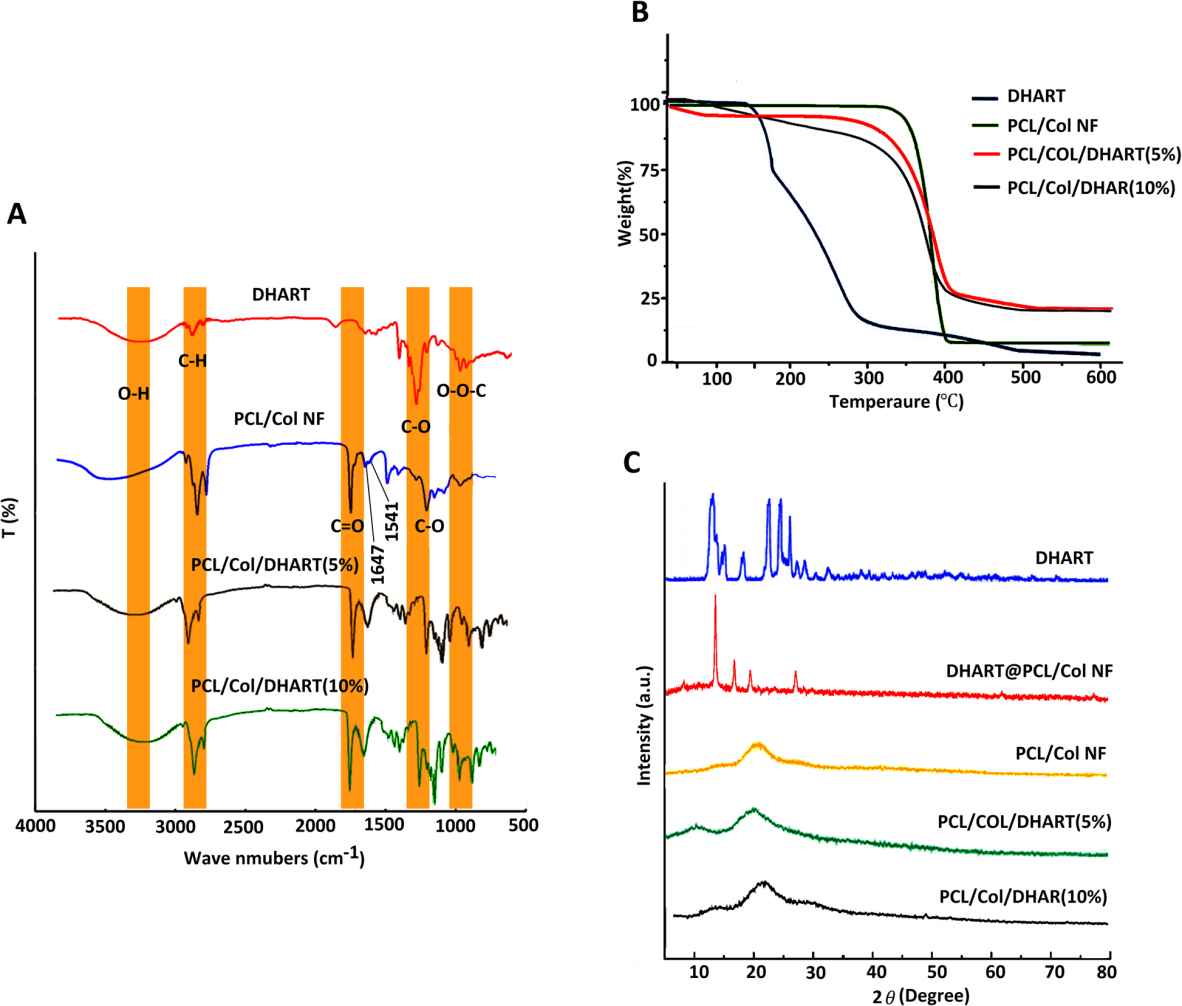
**A** FTIR spectra of DHART, PCL/Col, PCL/Col/DHART (5%), and PCL/Col/DHART (10%), **B** TGA curves of DHART, PCL/Col PCL/Col/DHART (5%), and PCL/Col/DHART (10%) NFs from 0 to 600 °C, and **C** XRD patterns of DHART, physically mixed DHART@PCL/Col, PCL/Col/DHART (5%), and PCL/Col/DHART (10%) NFs

The drug encapsulation process within NF carriers induced atypical shifts in FTIR spectroscopy. As a result, FTIR spectroscopic comparisons between pure DHART and PCL/Col/DHART NFs can be rather significant. Figure 2A displays the FTIR results of pure DHART, PCL/Col, and PCL/Col/DHART NFs. The therapeutic properties of DHART are attributed to its peroxide bridge, which can produce particularly potent oxidative stress [19]. The spectrum of pure DHART displayed typical bands at 3379 cm^−1^ (O–H stretching vibrations); 2947 cm^−1^ (C-H stretching); 1093 cm^−1^ (C-O stretching); 875 cm^−1^ (O–O-C stretching); 825 cm^−1^ (O–O stretching) presenting characters of O–O-C entityrespectively that characterizes 1,2,4-trioxanering [30]. In the PCL/Col NF spectra, the typical peaks at 1184 cm^−1^, 1220 cm^−1^, and 1727 cm^−1^ were assigned to the stretching band of C-O, C–O–C, and carboxylic acid functional groups of PCL, respectively. Col revealed the specific peaks at 1541 cm^−1^ (amide II) and 1647 cm^−1^ (amide I). As seen in Fig. 1, the presence of adsorption bands at 875, 1100 cm^−1^ in the pure DHART and PCL/Col/DHART NFs demonstrates the presence of DHART in its active state in PCL/Col/DHART NFs.

Thermal stability of DHART, PCL/Col, PCL/Col/DHART were evaluated through TGA. The detected initial weight loss for all samples up to 100 $^\circ{\rm C}$ is due to the moisture evaporation of compounds. DHART was initiated to decompose at 110 $^\circ{\rm C}$, monitored by loss of volatile substances of roughly 26% by weight to the second decomposition at 165 $^\circ{\rm C}$ [31]. In comparison, thermal degradation of PCL/Col, and PCL/Col/DHART NFs was between 389.70 and 424.65 °C, respectively (Fig. 2B).

To further characterization the interaction between DHART and PCL/Col, the crystal properties of the PCL/Col/DHART were examined by X-ray diffraction (Fig. 2 C) The diffractogram for DHART displays distinct peaks in the initial state, indicating the presence of crystalline phase [31], whereas the PCL/Col/DHART revealed a characteristic amorphous pattern. For the elimination of the possibility that the lower efficacy is due to the reduced weight percentage of DHART in the drug-loaded NF apparatus, the XRD analysis was performed on the physical mixture of PCL/Col/DHART with the equal quantity proportion. As shown in Fig. 2C, the DHART peak intensity is still evident in the physical mixture, demonstrating that its crystalline configuration is still evident. As a result, it demonstrates that the absence of the typical signal of DHART in PCL/Col/DHART NFs is not owing to the weak signal but instead to the amorphous structure. The current findings indicate that scaffold NFs can prevent DHART from the re-crystallizing process.

**Fig. 3:**
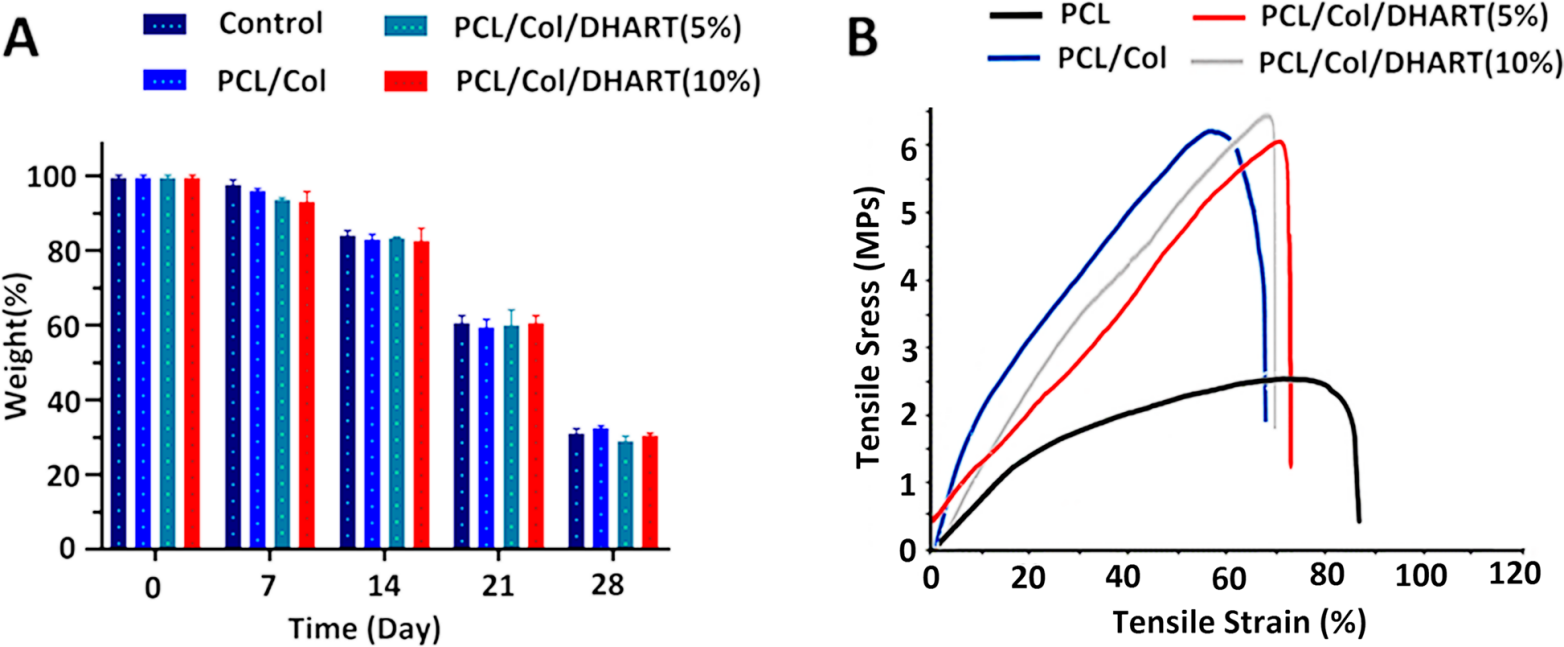
**A** Biodegradation profiles, **B** and typical tensile stress–strain curves of samples

Also, Fig. 3A shows the rate of degradation of the prepared NF scaffolds. The fiber weight reduction was not noticeable during the initial four days. After 28 days, more than 80% PCL/Col and PCL/Col/DHART NFs have degraded a relatively stable speed of weight reduction. Interestingly, no major differences in the removal percentage of PCL/Col and PLC/Col/DHART NFs were found. This was anticipated due to the small percentage of the total fiber weight that was related to DHART. After 28 days, the percentage of weight reduction for PCL/Col and PCL/Col/DHART NFs were 22.2% and 20.1%, respectively.

Besides, mechanical features must be considered in the efficient constructing scaffolds for tissue engineering and regenerative medicine applications. The characteristic tensile strain–stress diagrams of PCL, PCL/Col, and PCL/Col/DHART NFs were presented in Fig. 3B. Tensile stress for PCL, PCL/Col, PCL/Col/DHART (5%), and PCL/Col/DHART (10%) NFs were 2.33, 6.2, and 6.14, 6.26 MPa and can bear a strain of 86, 66, 68 and 70%, respectively. The Young’s modulus value for PCL, PCL/Col, PCL/Col/DHART (5%), and PCL/Col/DHART (10%) NFs were 11.21, and 29.13, 30.2, 31.7 MPa. The result displayed greater tensile stress–strain values for PCL/Col than PCL NFs due to enhanced mechanical characteristics. The findings suggested that combining PCL with Col improves the mechanical characteristics of NF scaffolds [32]. Also, as shown in this diagram, the tensile mechanical and physical properties of DHART-loaded NFs and neat NFs were remarkably similar, demonstrating that incorporation of DHART molecules into the PCL/Col NFs could not significantly change the fiber's mechanical properties.

### Drug release assay

**Fig. 4:**
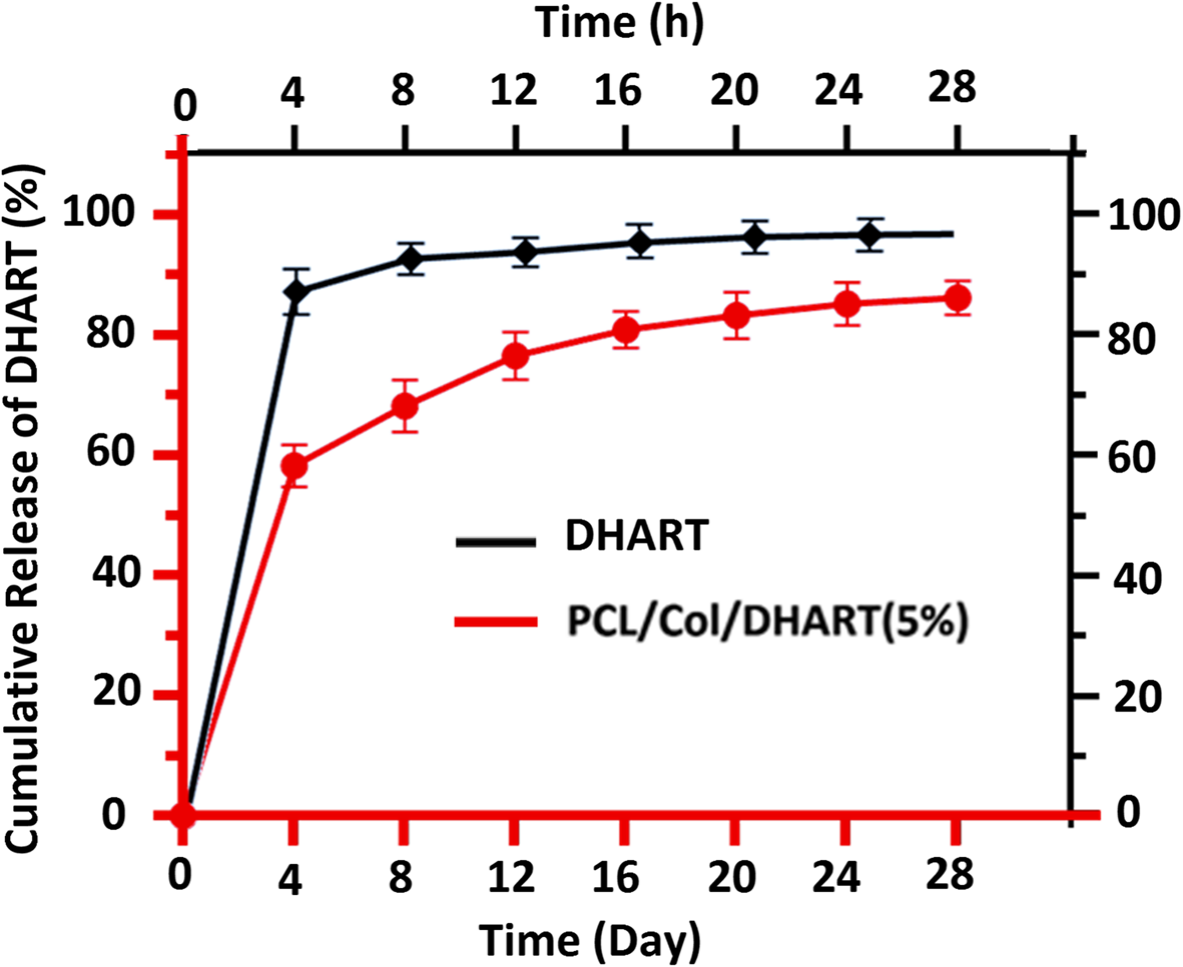
Cumulative DHART release in PBS (pH 7.4) at regular intervals. The data are presented as mean ± SD (*n* = 3)

Cumulative DHART release from PCL/Col/DHART (5%) NFs was evaluated for 21 days at 37 °C in PBS solution. The release profile of PCL/Col/DHART (5%) NFs is shown in Fig. 4. As shown in Fig. 4, PCL/Col/DHART (5%) NFs with the highest content of PCL demonstrated that fast drug release at first, then slower sustained release, which is typical of reservoir-type scaffolds. DHART burst release from membranes was first identified in the early days. PCL/Col/DHART NFs displayed a more constant release of DHART after 4 days. The burst release phase was due to the rapid release of amorphous DHART from the PCL/Col/DHART surfaces. The DHART encapsulated within the PCL/Col NFs dispersed slowly onto the NF surface and then into the medium solution, providing a gradual rate of diffusion. Free DHART control exhibited the typical rapid release, with > 85% DHART released within 4 h at pH 7.4. However, PCL/Col/DHART released about 87% of DHART after 2 weeks of incubation. This is explained by interactions between DHART and PCL/Col hybrid polymer, which resulted in a significant enhancement in the sustained release of drugs from the PCL/Col/DHART (5%) NFs.

These findings are in accordance with those from a study by Huo P and colleagues, demonstrating that the sustained release of Artemisinin (ART) from PCL/Col NFs. They reported that these molecules diffuse into the PBS medium from the surface of the PCL/Col NFs and form holes on the surface of the NFs. As the release progresses, pores are gradually formed inside the NFs, and the medium gradually penetrates the NFs. Subsequently, the ART embedded in the PCL/Col NFs gradually dissolves and eventually completely dissolves in the medium, leaving the NF scaffolds (19).

### The viability and proliferation of hADSCs on PCL/Col/DHART NFs

**Fig. 5:**
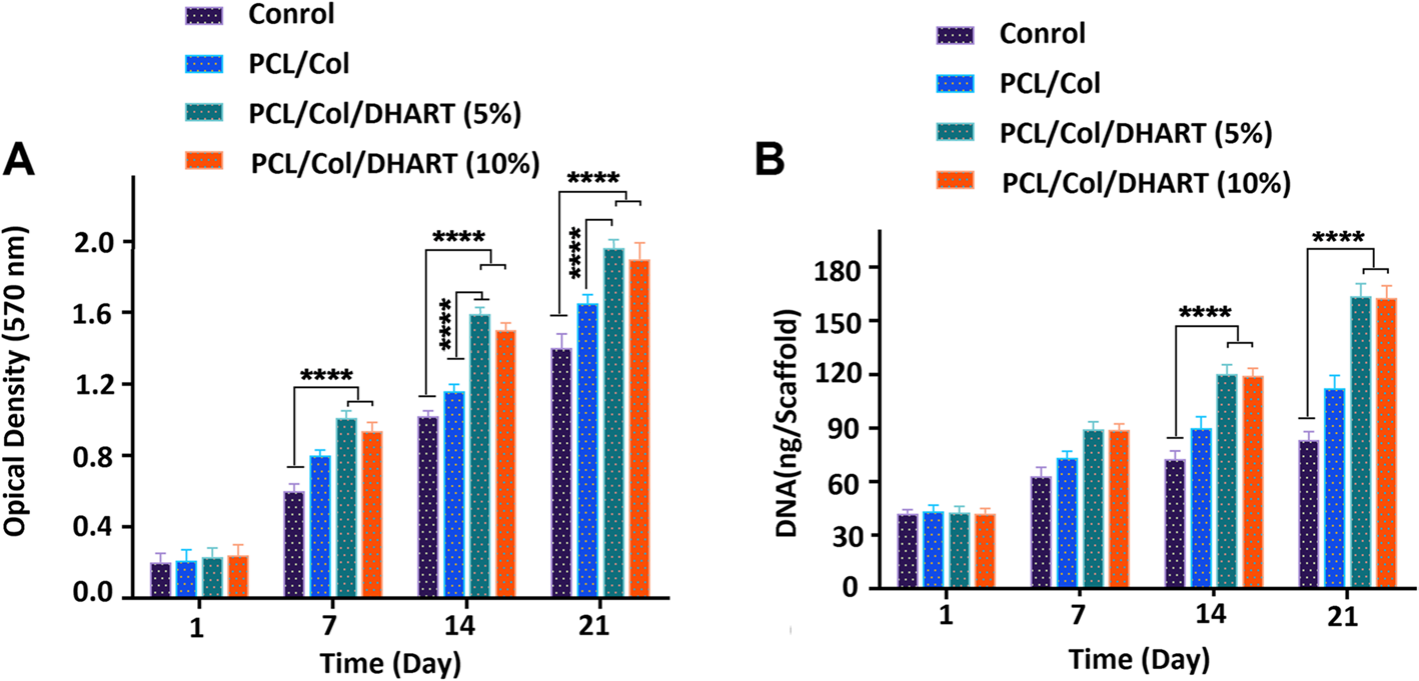
The viability and proliferation of hADSCs on the NFs evaluated by MTT and PicoGreen assays, respectively. ****P < 0.0001 vs. control was considered significant. Results are mean ± SD (*n* = 3)

hADSCs were seeded on the prepared NFs and their cytocompability and the viability and proliferation of these cells were evaluated using MTT assay at 1-, 7-, 14-, and 21- days (Fig. 5A). The results revealed that the viability and proliferation improved in all of the treated samples and there was no important difference in the viability of the PCL/Col treated group for different time intervals of the experiment. However, it is observed that the viability of PCL/Col/DHART treated groups was significantly higher on days 14 and 21 than for the neat PCL/Col treated groups. Importantly, it was found that the PCL/Col/DHART (5%) treated NFs had higher cell viability than PCL/Col/DHART (10%) treated NFs for different time intervals of the experiment, obviously on day 21 (*p* < 0.0001). The results demonstrated that PCL/Col/DHART (5%) NFs are nontoxic and could significantly improve the viability of hADSCs.

For the evaluation of the amount of cell proliferation, PicoGreen examine was used to determine the DNA quantity in the treated NF scaffolds (Fig. 5B). There was no considerable variance in the DNA quantity at periodic intervals for the control and PCL/Col treated groups. However, PCL/Col/DHART treated groups had a higher DNA value after 14 and 21 days. In comparison to the other groups, the DNA quantity in the PCL/Col/DHART (5%) treated NFs significantly increased from day 1 to day 21 (*p* < 0.0001). It is expected that the prolonged release of DHART from the NF scaffolds provides long-term attachment for the hADSCs viability and proliferation. These findings are in accordance with those from a study by Mashayekhi et al., demonstrating that the sustained release of DHART from NFs increase the adhesion, viability, and proliferation of hADSCs.

### The adhesion and proliferation of hADSCs on PCL/Col/DHART NFs

**Fig. 6:**
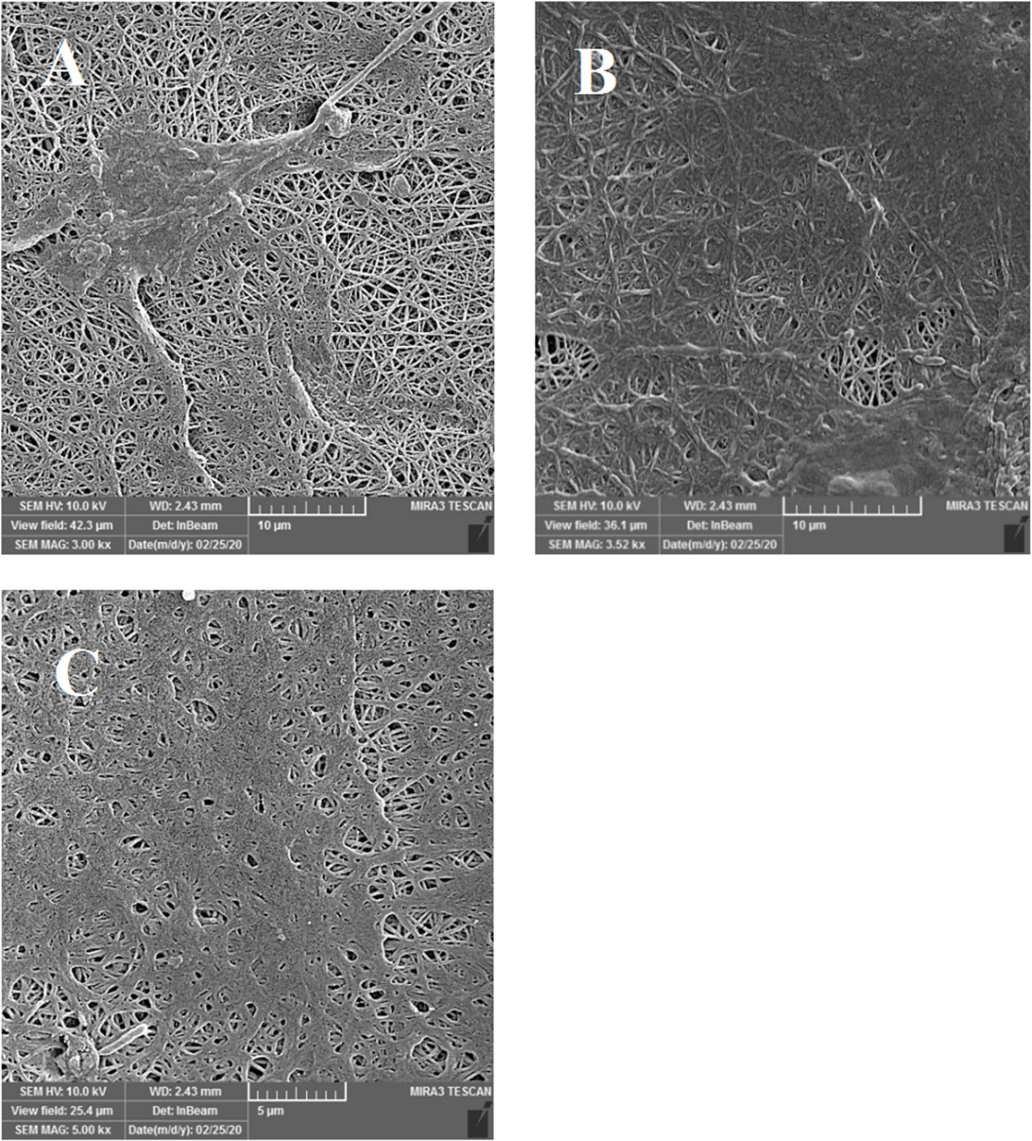
FE-SEM images of adhesion of hADSCs on the **A** PCL/Col, **B** PCL/Col/DHART (5%), **C** PCL/Col/DHART (10%) groups after 21 days of cell seeding

The FE-SEM analysis showed the morphology and distribution of the treated NF groups after 21 days of incubation (Fig. 6). These results indicated that cell lines cover the surface of all the NFs and can adhere to them. After 21 days of culture, the PCL/Col/DHART treated groups revealed better attachment and proliferation compared to PCL/Col treated NFs. The most distribution and covering of hADSCs were found on PCL/Col/DHART (5%) treated group, demonstrating high biocompatibility and non-toxicity of PCL/Col/ DHART (5%) for hADSCs. These results were compatible with the detected results of the viability and proliferation of hADSCs on PCL/Col/DHART NFs.

### The expression levels of osteogenic-specific genes of hADSCs on PCL/Col/DHART NFs

**Fig. 7:**
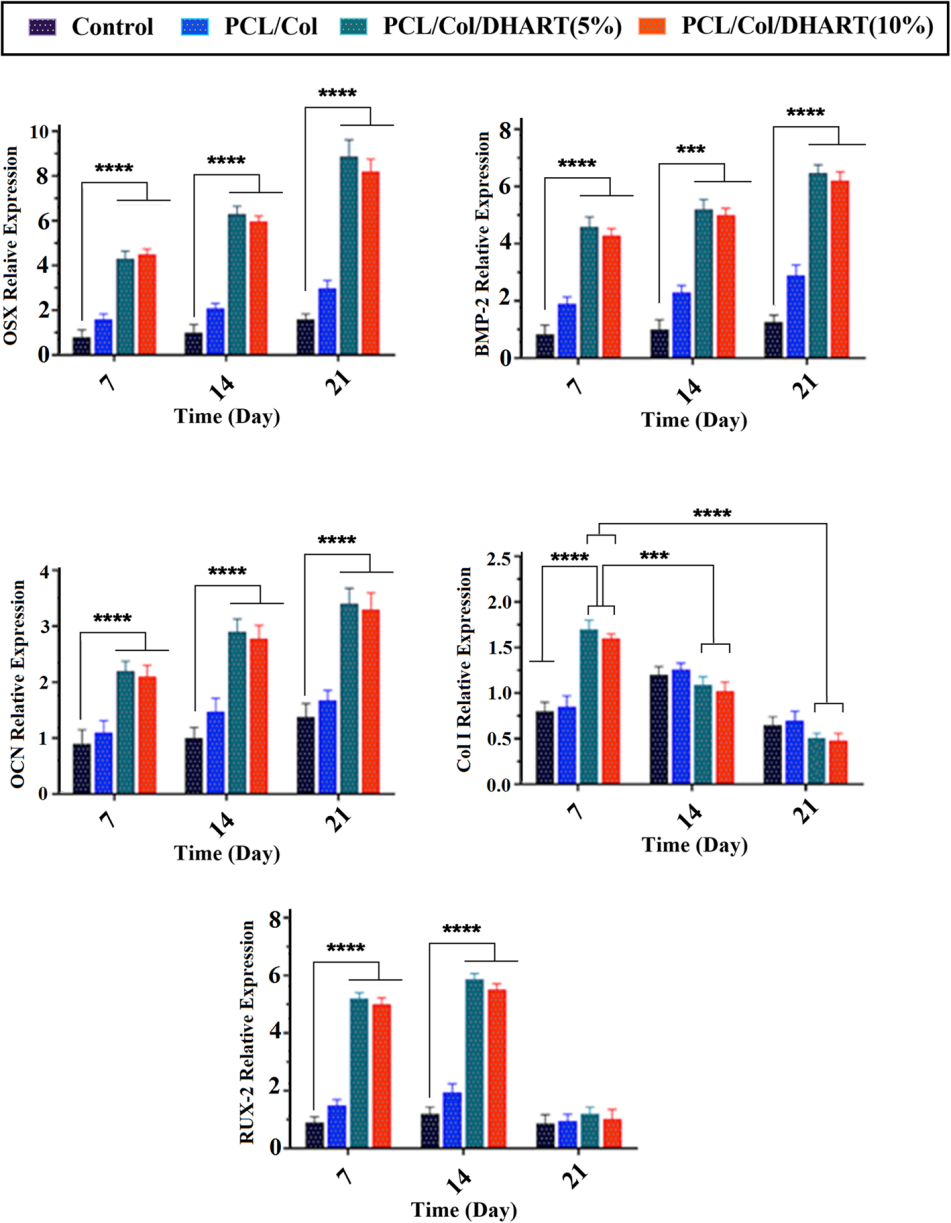
mRNA expression levels of OSX, BMP-2, OCN, Col I, and RUNX-2, in hADSCs seeded on the NFs after 7-, 14-, and 21- days of culture. The GAPDH gene was used as an internal control. *P* < 0. 05 vs. control was considered significant. Results are mean ± SD (*n* = 3). *** represents *p* ≤ 0.001, **** represents *p* ≤ 0.0001

For the evaluation of the proliferation and differentiation of treatment groups, the comparative mRNA expression of major osteoblast differentiation factors such as OSX, BMP-2, RUNX-2, Col I, and OCN were assessed by real-time PCR analysis (Fig. 7).

Col I is an initial osteoblast differentiation factor that helps to generate the external collagen network, which is necessary for the formation of the osteoblastic appearance [33]. The Col I expression peak was higher in DHART-loaded NFs on day 7, but it was reduced on day 14. Also, previous studies have revealed that Col I is up-regulated during the osteoblastic differentiation early phases and down-regulated during the later phases. Also, Runx2 is another initial key factor for osteoblastic differentiation, which up-regulates OCN fundamental bone matrix factor [34]. The expression level of Runx2 mRNA in the treated scaffolds increased until the 14 days and then decreased in all of the treated groups (Fig. 7). The expression level of Runx2 mRNA was rather higher in the DHART-loaded NF (5%) groups compared with the other treated groups.

OSX, BMP-2, and OCN are the most prominent non-collagenous bone formation factors that show a critical role in the early stages of bone formation calcification and are widely used as the late factor in bone formation differentiation [35]. As anticipated, the expression mRNA level of these proteins increased dramatically over time, with the greatest value expression observed on day 21 for the DHART and PCL/Col/DHART (5%) (Fig. 6). Previous studies have suggested that DHART plays an unknown mechanism in the control of human mesenchymal stem cells (hMSCs) osteogenic differentiation and proliferation. Licheng Ni, et al. reported DHART’s effect on hMSCs’ osteogenic differentiation and proliferation, together with its fundamental metabolic pathways. They found that DHART had no effect on the hMSC proliferation but improved osteogenic differentiation. It most possibly carried out its activity via the ERK1/2 and Wnt/β signaling pathways [23].

### Osteoblastic differentiation on PCL/Col/DHART NFs

**Fig. 8:**
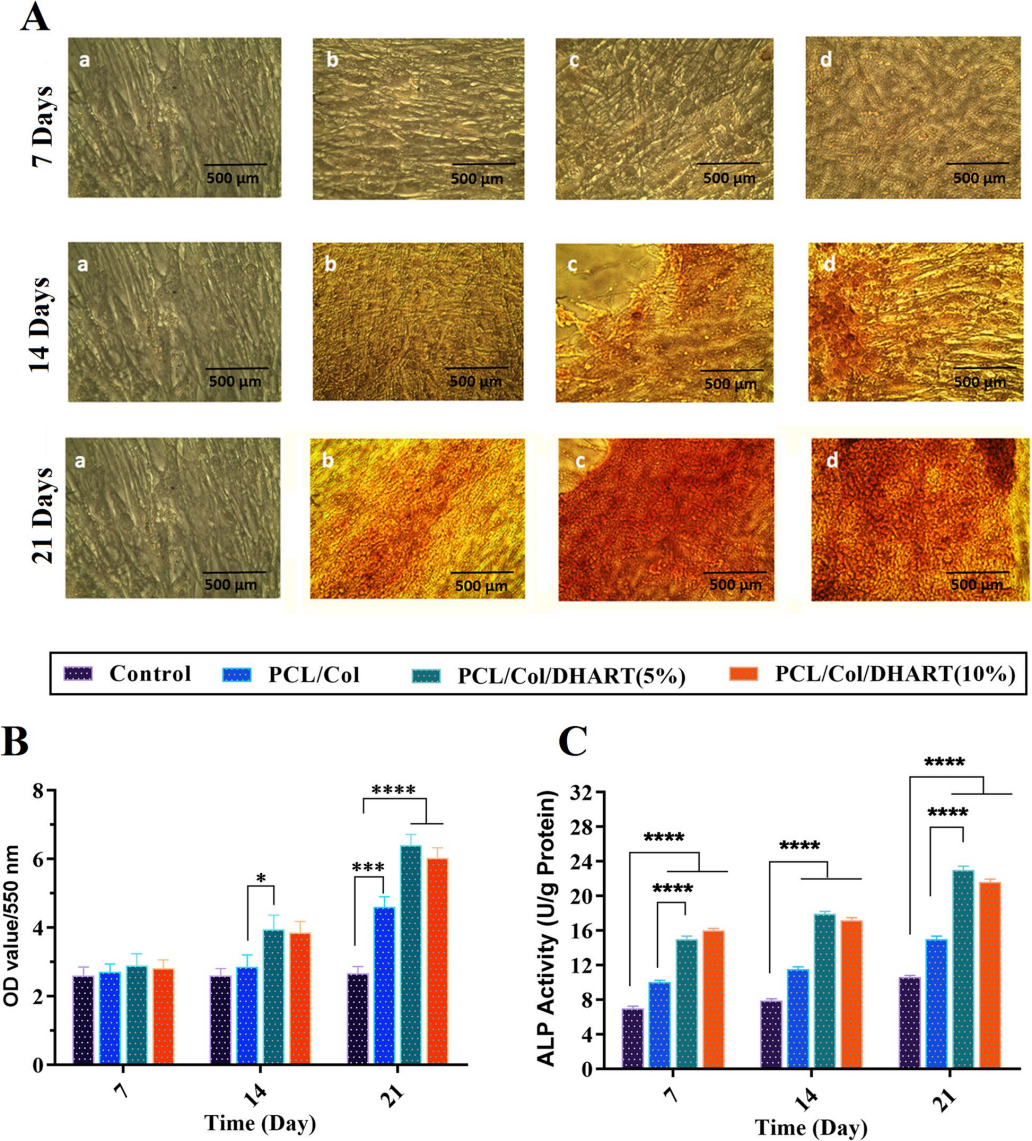
**A** Alizarin red staining of hADSCs seeded on the treatment groups of **a**) control, **b**) PCL/Col, **c**) PCL/Col/DHART (5%) and **d**) PCL/Col/DHART (10%) at 7-, 14- and 21- days after osteogenic differentiation, and **B** in quantitatively colorimetric data of alizarin red staining. Results are mean ± SD (*n* = 3). **C** The ALP activity of hADSCs seeded on the treatment groups of control, PCL/Col, PCL/Col/DHART (5%) and PCL/Col/DHART (10%) at 7-, 14- and 21- days after osteogenic differentiation. *P* < 0. 05 vs. control was considered significant. Results are mean ± SD (*n* = 3). * represents *p* ≤ 0.05, *** represents *p* ≤ 0.001, **** represents *p* ≤ 0.0001

The most common procedures for assessing stem cell osteoblastic development on platforms are the evaluation of total calcium concentration and ALP activity. Alizarin Red staining was performed to visualize calcium deposition in osteoblasts at 7-, 14- and 21- days following the induction of osteogenesis. (Fig. 8A and B). It was found that calcium deposition in osteoblasts was significantly increased, and these promoting effects were time-dependent. These results are in accordance with those from a study by Xia T et al. [36]. After 24-days incubation, the results of mineralization demonstrated that the treated NF scaffolds had increased calcium deposition compared to the control-treated group. The calcium deposited quantity on the DHART-loaded NFs was found to be much higher than that on control, and neat NF treated scaffolds due to the biofunctionality of released DHART which stimulated the osteogenic differentiation. Moreover, the osteoblast phenotype was evaluated by determining ALP activity. The results revealed that ALP activity was also increased in a time-dependent manner (Fig. 8C). In comparison to the control-treated group, PCL/Col, and PCL/Col/DHART NF scaffolds considerably improved ALP activity on the 21 days. Particularly, it was found that PCL/Col/DHART (5%) treated groups considerably enhanced the cell ALP activity.

## Conclusion

In the current study, DHART-loaded PCL/Col electrospun NF scaffolds were manufactured to examine the effect of DHART to improve the osteoblastic differentiation ability of hADSCs. DHART re-crystallization can be considerably inhibited within the electrospun PCL/Col NF system, and sustained release effectiveness can be maintained by regulating the percentage of PCL/Col hydrophobic/hydrophilic polymers. The mechanical properties, as well as the proliferation rate of hADSCs, were greatly improved by DHART-loaded PCL/Col NFs. Furthermore, the high expression of osteoblastic markers and enhanced ALP activity revealed that the produced PCL/Col/DHART (5%) NF scaffold could provide a possible advantage for hADSCs osteoblastic differentiation.

## Data Availability

Please contact author for data requests.
